# Adjuvant Hormonotherapy and Cardiovascular Risk in Post-Menopausal Women with Breast Cancer: A Large Population-Based Cohort Study

**DOI:** 10.3390/cancers13092254

**Published:** 2021-05-08

**Authors:** Matteo Franchi, Roberta Tritto, Luigi Tarantini, Alessandro Navazio, Giovanni Corrao

**Affiliations:** 1 National Centre for Healthcare Research and Pharmacoepidemiology, 20126 Milan, Italy; roberta.tritto@unimib.it (R.T.); giovanni.corrao@unimib.it (G.C.); 2 Laboratory of Healthcare Research & Pharmacoepidemiology, Department of Statistics and Quantitative Methods, University of Milano-Bicocca, 20126 Milan, Italy; 3 Cardiology Division, Presidio Ospedaliero ASMN Azienda USL Reggio Emilia-IRCCS, 42123 Reggio Emilia, Italy; luigi.tarantini@ausl.re.it (L.T.); alessandro.navazio@ausl.re.it (A.N.)

**Keywords:** breast cancer, aromatase inhibitors, cardiovascular risk, heart failure, clinical practice

## Abstract

**Simple Summary:**

For post-menopausal women with estrogen-receptor-positive breast cancer, treatment with aromatase inhibitors reduces disease recurrence and mortality, as compared to tamoxifen. Nevertheless, women treated with aromatase inhibitors more often develop hyperlipidemia, hypercholesterolemia and hypertension, which are recognized cardiovascular (CV) risk factors. Concerns about CV safety of aromatase inhibitors had been raised by several studies. Our results showed that adjuvant therapy with aromatase inhibitors of breast cancer women is associated with increased risk of heart failure and combined CV events, and such therapy probably amplifies the “intrinsic” CV risk of the patient. They underline the importance of interdisciplinary collaboration between oncologists and cardiologists in evaluating the risk/benefit ratio of the choice of hormone therapy.

**Abstract:**

Background: Whether aromatase inhibitors (AIs) increase the risk of cardiovascular (CV) events, compared to tamoxifen, in women with breast cancer is still debated. We evaluated the association between AI and CV outcomes in a large population-based cohort of breast cancer women. Methods: By using healthcare utilization databases of Lombardy (Italy), we identified women ≥50 years, with new diagnosis of breast cancer between 2009 and 2015, who started adjuvant therapy with either AI or tamoxifen. We estimated the association between exposure to AI and CV outcomes (including myocardial infarction, ischemic stroke, heart failure or any CV event) by a Cox proportional hazard model with inverse probability of treatment and censoring weighting. Results: The study cohort included 26,009 women starting treatment with AI and 7937 with tamoxifen. Over a median follow-up of 5.8 years, a positive association was found between AI and heart failure (Hazard Ratio = 1.20, 95% CI: 1.02 to 1.42) and any CV event (1.14, 1.00 to 1.29). The CV risk increased in women with previous CV risk factors, including hypertension, diabetes and dyslipidemia. Conclusions: Adjuvant therapy with AI in breast cancer women aged more than 50 years is associated with increased risk of heart failure and combined CV events.

## 1. Introduction

Five-year adjuvant treatment of women with breast cancer positive for estrogen receptors (ERs) with tamoxifen reduces breast cancer mortality of about one third every year, irrespectively from age, use of chemotherapy, progesterone receptor status, or other tumor characteristics [1]. Further reductions in disease recurrence and mortality had been reported for post-menopausal ER positive breast cancer women treated with aromatase inhibitors, as compared to tamoxifen alone [2,3]. Nevertheless, aromatase inhibitors reduce estrogen concentrations and therefore the estrogen-mediated protective effects on cardiovascular system, such as regulation of serum lipid metabolism and vasodilation. Women treated with aromatase inhibitors, indeed, compared with patients who receive tamoxifen, are more likely to develop hyperlipidemia, hypercholesterolemia, and hypertension, which are recognized cardiovascular (CV) risk factors [4]. Moreover, concerns about CV safety of aromatase inhibitors had been raised by meta-analyses of randomized clinical trials [5,6]. Few and inconsistent observational studies had been carried out on this topic. Indeed, while some investigations showed that women treated with aromatase inhibitors had significant increase of myocardial infarction [7], heart failure [8], CV mortality [8] and other CV outcomes [9], other observational studies did not find any association with myocardial infarction [8,9,10,11] and stroke [8,9,10]. Based on these premises, we carried out a large retrospective population-based cohort study for comparing CV risk in a very large real-world cohort of breast cancer women on treatment with aromatase inhibitors or tamoxifen.

## 2. Materials and Methods

### 2.1. Data Source and Target Population

Data were retrieved from the healthcare utilization databases of Lombardy, a region of Northern Italy accounting for about 16% (almost ten million) of its entire population. The Italian National Health Service (NHS) provides universal and free of charge healthcare services, including those for cancer care. In Lombardy, an automated system of databases collecting a variety of information allows management of healthcare services. Details on information included in the healthcare utilization databases of Lombardy and their use in the field of cancer have been reported elsewhere [12]. Specific diagnostic and therapeutic codes used for the current study are given in supplementary material (Table S1).

The target population included all women who, during the years 2009 to 2015, were resident in Lombardy and beneficiaries of the NHS and had at least one hospital admission for breast cancer. The date of the first hospital admission for breast cancer occurred during this period was defined index date. We excluded women who (i) at the index date were younger than 50 years (age ≥ 50 was used as a surrogate of postmenopausal status); (ii) at the index date or in the following six months did not receive breast surgery (in order to identify a more homogenous cohort of breast cancer women); (iii) during the five-year period prior the index date had CV-related or cancer-related hospital admission or oncologic medicaments, including hormonotherapy (in order to include incident cancer cases without previous CV diseases and new hormonotherapy users); (iv) during the six months following the index date had signs of distant metastasis (in order to exclude women with advanced stage of the disease). Among the remaining women, those who started therapy with either aromatase inhibitors (including anastrozole, letrozole and exemestane) or tamoxifen within one year from surgery were included in the final study cohort. We labelled the date of first prescription of hormonotherapy as “treatment start”.

### 2.2. Exposure Definition

We used two approaches for defining exposure. First, with the intention-to-treat approach, cohort members accumulated person-years of exposure to either aromatase inhibitors or tamoxifen (according to medicament initially dispensed) from the date of treatment start until the earliest date among t outcome occurrence (see below), death, migration or 31 December, 2019 (end-point of follow-up). Second, for taking into account possible changes in therapeutic strategy during follow-up, with the as-treated approach, each woman was considered exposed to either aromatase inhibitors or tamoxifen until she switched from aromatase inhibitors to tamoxifen, or vice versa, being information censured at the earliest date among outcome occurrence, switching, death, migration or 31 December, 2019.

### 2.3. Outcome Ascertainment

We separately recorded hospital admissions for myocardial infarction, heart failure, ischemic stroke, or any CV event, the latter being defined CV composite outcome. We labelled the date of the first hospitalization for each of these causes as “outcome occurrence”.

### 2.4. Additional Measurements

We considered baseline characteristics of cohort members including those measured at treatment start (age and calendar year), comorbidities detected through in-hospital diagnoses experienced in the 3-year period prior starting therapy (peripheral vascular disease, venous thromboembolism, chronic obstructive pulmonary disease and chronic kidney disease), and selected co-treatments dispensed in the year prior treatment start (anticoagulants, antihypertensive, antithrombotic, statins, antidiabetic, nonsteroidal anti-inflammatory drugs, opioids, bisphosphonates, antidepressants and hormone replacement medicaments). Chemotherapy and radiotherapy dispensed between index date and treatment start were recorded.

We also recorded time-varying characters, including hospital admissions, outpatient services (i.e., visits, diagnostic procedures, and laboratory tests) and cardiac medicaments dispensed from treatment start until the switch of therapy.

Finally, according to previous treatment with statins, antihypertensive, and antidiabetic drugs, we stratified patients into three CV risk profile: low-risk (i.e., no CV risk factors), medium-risk (i.e., one CV risk factors), and high-risk (i.e., two or more CV risk factors).

### 2.5. Data Analyses

We compared patients starting treatment with aromatase inhibitors or tamoxifen in terms of standardized differences of baseline characters (we considered negligible those differences with absolute standardized differences < 0.10) [13], as well as the occurrence of each of the outcome above mentioned. We measured the latter by means of incidence rates (and Poisson-based 95% confidence intervals, CI) and Kaplan–Meier cumulative incidence curves, and we tested between-group differences according to the test of homogeneity of incidence rates and the log-rank test, respectively [14]. We used the Cox proportional hazard model for estimating the hazard ratio (HR), and corresponding 95% CI, for the exposure–outcome association.

To minimize confounding by indication, we developed a propensity score (PS) matching design [15]. We estimated the PS by regressing all the baseline covariates towards medicament employed at starting treatment (i.e., aromatase inhibitors or tamoxifen) with a logistic regression model. Each cohort member on aromatase inhibitors was then 1:1 matched with a woman randomly selected from cohort members on tamoxifen with similar PS (tolerating a difference of ±0.01).

As independence between switching and outcome is not ensured by the as-treated approach, that is, the reasons of switching may be related to the outcome onset so generating estimates biased by informative censoring, the exposure–outcome association was further estimated by means of a Cox proportional hazards model with inverse probability of treatment and censoring weights. The inverse probability of censoring weighting (IPCW) method consists of censoring observations at the time of switching therapy and assigning to each observation a weight inversely proportional to the probability of censoring, conditional to covariates measured until censor. Weights were generated by fitting a time-dependent Cox regression model to estimate the probability of censoring due to switching therapy. The model, fitted separately among patients initially treated with either aromatase inhibitors or tamoxifen, included the time-varying characters measured from treatment start until switching. The censoring weights were then stabilized by the probability of censoring conditional on the treatment received (i.e., aromatase inhibitors or tamoxifen) [16].

In addition, to minimize confounding by indication, the inverse probability of treatment weighting (IPTW) method was used. Weights were estimated using a logistic regression model to determine the probability of receiving aromatase inhibitors versus tamoxifen, conditional on variables measured at or before cohort entry. The treatment weights were stabilized by the probability of the treatment received (i.e., the prevalence of exposure to either aromatase inhibitors or tamoxifen).

The product of the two stabilized weights (IPC and IPT) was used as final weights to estimate the parameters of the marginal Cox proportional hazard model for estimating the exposure–outcome association. Final weights were truncated at 1st and 99th percentile of their distribution, to reduce their variability [17]. Since, after weighting, study groups were unbalanced with respect to patients’ age, the model also included age as covariate.

For all the tested hypotheses, two-tailed *p*-values less than 0.05 were considered statistically significant.

## 3. Results

### 3.1. Study Cohort

**Figure 1:**
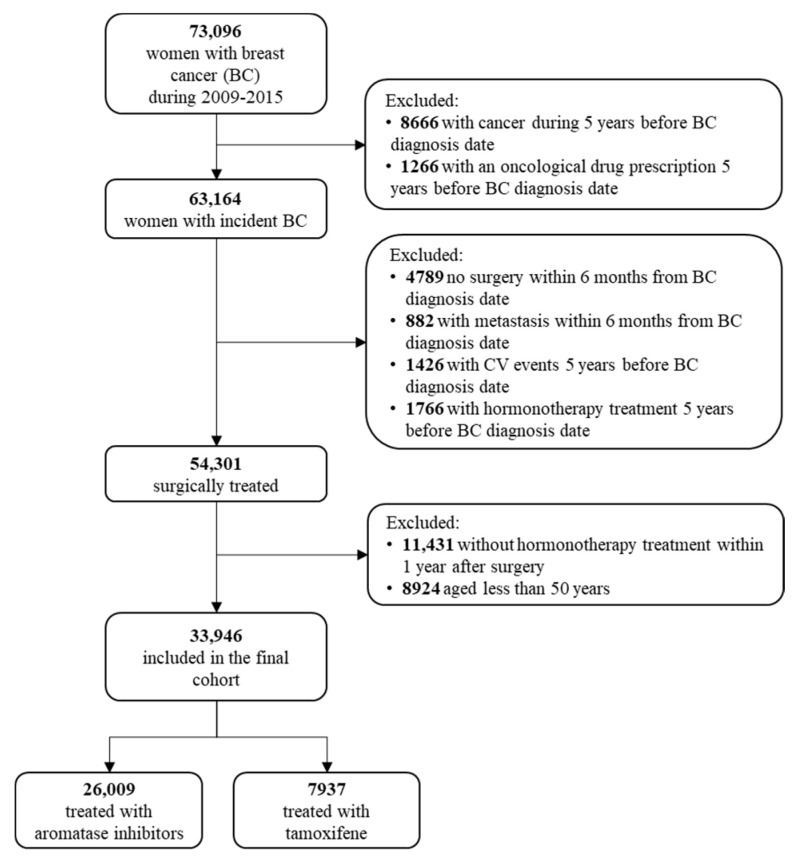
Flow chart of inclusion and exclusion criteria in the final cohort study.

The process of the selection of the study cohort is reported in Figure 1. Among the 73,096 women with a diagnosis of breast cancer during the period 2009–2015, 33,946 met the inclusion criteria. Of these, 26,009 (76.6%) started adjuvant therapy with aromatase inhibitors and the remaining 7937 (23.4%) with tamoxifen. Baseline characteristics of women included in the study cohort are reported in Table 1. In the original (unmatched) cohort, the prevalence of comorbidities was low, likely because the exclusion of women with previous clinically relevant CV requiring hospital admission. Women treated with aromatase inhibitors were older, had higher prevalence of use of statins, anticoagulants, antidiabetic, antihypertensive and antithrombotic drugs, and were more likely to receive chemotherapy. Baseline characteristics were well balanced between treatments in the PS-matched cohort, as well as in the IPCW weighted cohort (with the only exception for age in the latter cohort).

### 3.2. Composite CV Outcome

Overall, 473 and 428 hospital admissions with any CV diagnosis were observed among PS-matched women who respectively started hormonotherapy with aromatase inhibitors and tamoxifen. The corresponding incidence rates of 9.03 (95% CI: 8.22 to 9.85) and 7.96 (7.21 to 8.71) events every 1000 person-year (Table 2) were significantly different (*p* = 0.029). There was no evidence that the type of hormonotherapy employed at starting treatment affected the risk of at least one CV event when observations were censored any time the switching occurred. Conversely, a significant 14% excess of CV hospital admissions was observed for both the intention-to-treat approach and the IPCW analysis (Table 3).

### 3.3. Myocardial Infarction

**Figure 2:**
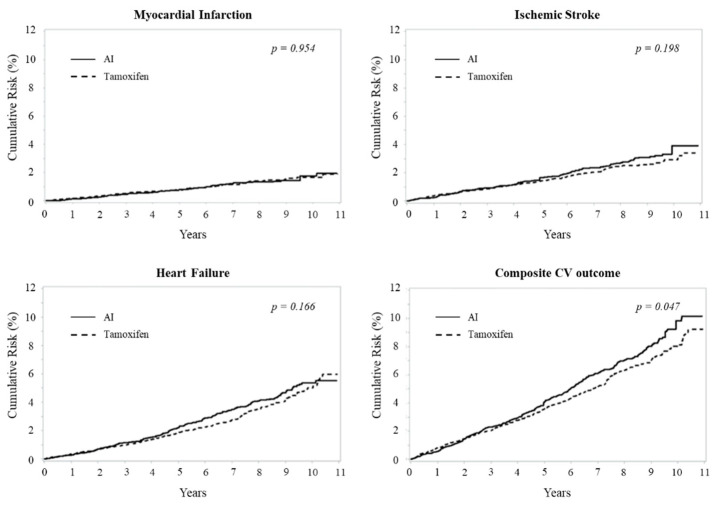
Kaplan–Meier estimates of the cumulative risk in patients with breast cancer treated, respectively, with aromatase inhibitors (AI) and tamoxifen. Cumulative risk over an 11-year horizon of any cardiovascular outcome (**top left**), myocardial infarction (**top right**), ischemic stroke (**bottom left**) and heart failure (**bottom right**) based on an intention-to-treat approach in which cohort women were classified on the therapy (aromatase inhibitors or tamoxifen) initially received. Patients were 1:1 matched by propensity score.

Overall, 89 and 92 hospitalizations for myocardial infarctions were observed among PS-matched women who respectively used aromatase inhibitors and tamoxifen, the corresponding rates incidence being 1.67 (1.32 to 2.02) and 1.69 (1.34 to 2.03) events every 1000 person-year (*p* = 0.470) (Table 2). Cumulative incidences were also remarkably similar, being 1.7% of women belonging to both the groups those who experienced myocardial infarction after 11 years from treatment start (*p* = 0.954) (Figure 2). Finally, there was no evidence that the type of hormonotherapy employed at starting treatment affected the risk myocardial infarction, irrespectively whether an intention-to-treat approach was employed, observations were censored anytime the switching occurs, and observations were weighed by the IPCW analysis (Table 3).

### 3.4. Ischemic Stroke

Overall, 182 and 163 hospitalizations for ischemic strokes were observed among PS matched women who respectively started with aromatase inhibitors and tamoxifen, the corresponding incidence rates being 3.43 (2.93 to 3.93) and 3.00 (2.54 to 3.46) events every 1000 person-year (*p* = 0.106) (Table 2). Cumulative incidence did not differ between groups during the first 5 years after treatment start, but slightly increased afterwards among users of aromatase inhibitors, reaching 3.9% and 2.9% at the end of follow-up (*p* = 0.198) (Figure 2). Finally, analogously to myocardial infarction, for ischemic stroke also there was no evidence that the type of hormonotherapy at the starting treatment affected the risk of ischemic stroke for any of the employed designs (Table 3).

### 3.5. Heart Failure

Overall, 266 and 243 hospitalizations for heart failure were observed among PS-matched women who respectively started with aromatase inhibitors and tamoxifen. The corresponding incidence rates were 5.02 (4.41 to 5.62) and 4.47 (3.91 to 5.03) events every 1000 person-year (*p* = 0.097) (Table 2). Cumulative incidence did not differ between groups during the first 4 years after treatment start, but slightly increased afterwards among users of aromatase inhibitors, reaching 5.4% and 5.0% at the end of follow-up (*p* = 0.166) (Figure 2). Finally, there was no evidence that the type of hormonotherapy employed at starting treatment affected the risk of heart failure when the intention-to-treat approach was employed and when observations were censored anytime the switching occurred. Conversely, a significant 20% (2–42%) excess of heart failure was observed from the IPCW analysis (Table 3).

### 3.6. Heart Failure According with CV Risk Factors

**Figure 3:**
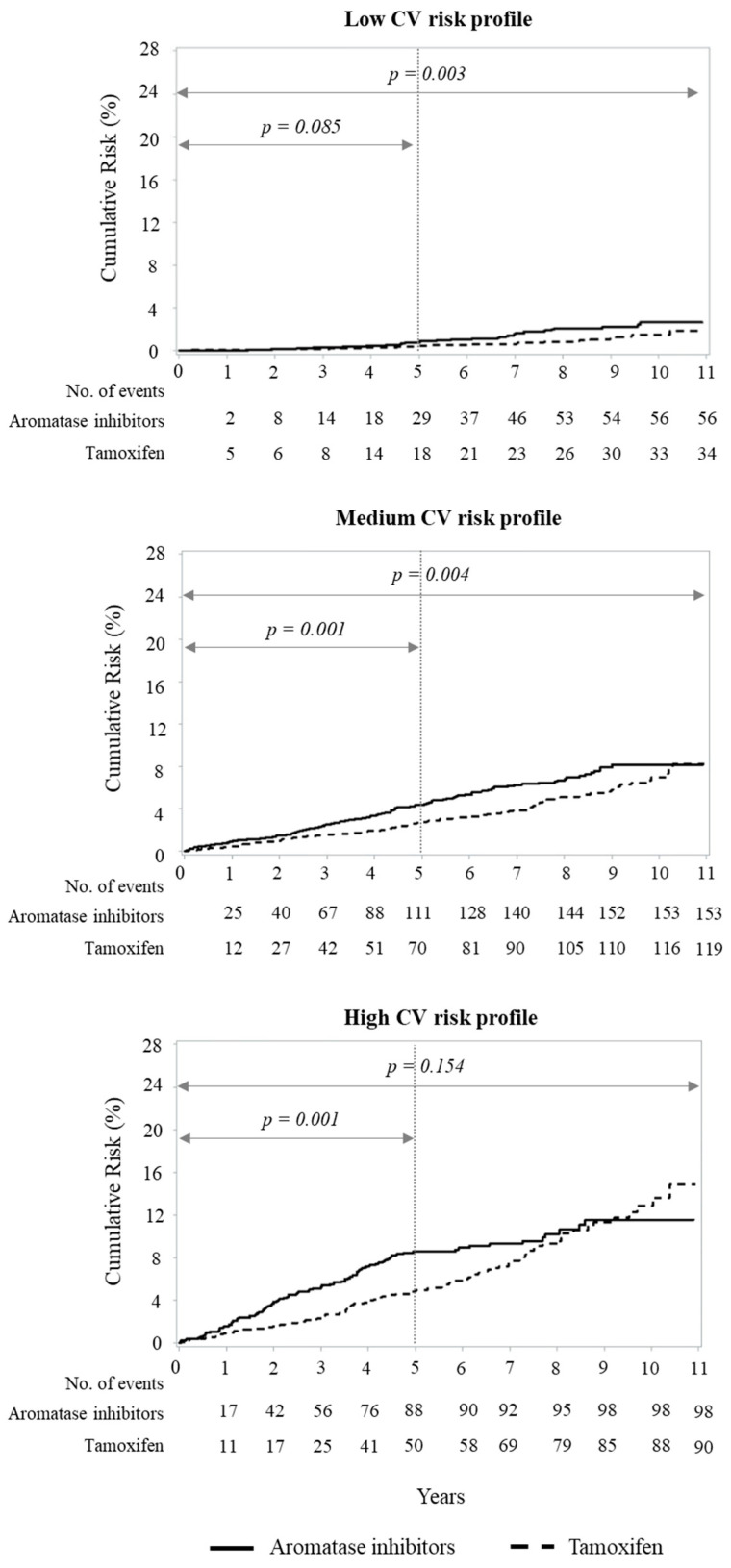
Kaplan–Meier estimates of the cumulative risk of heart failure in patients with breast cancer treated, respectively, with aromatase inhibitors (AI) and tamoxifen stratified by baseline CV risk. Cumulative risk over an 11-year horizon of heart failure in low CV profile (**top**), medium CV profile (**central**), and high CV profile (**bottom**) based on an intention-to-treat approach in which cohort women were classified on the therapy (aromatase inhibitors or tamoxifen) initially received. Patients were 1:1 matched by age at surgery.

Matched cohort patients exhibited the following distribution of CV risk factors: 8136 (51.3%) patients had no CV risk factor (low risk), 5516 (34.8%) patients had only one CV risk factor (medium risk) and 2212 (13.9%) patients had two or more CV risk factors (high risk). As expected, during the follow-up, hospitalizations for heart failure occurred more frequently in the high-risk group (8.5%), compared to the medium-risk group (4.9%) and the low-risk group (1.1%) (*p* < 0.001). The time trend analysis showed a different course of heart failure occurrence in the two treatment groups, suggesting an interaction between the hormone therapy and the patient’s CV risk profile (Figure 3). During the 5-year treatment period, women with low CV risk did not show significant differences in heart failure according with the type of anti-estrogen therapy received (*p* = 0.085). However, continuing observation after the end of treatment, the aromatase inhibitors therapy was associated to an increased risk (HR = 1.88 (1.22–2.88), *p* = 0.003). Conversely, in high-risk patients, aromatase inhibitor therapy resulted in a significant increase in heart failure early during the treatment period (HR = 1.82 (1.29–2.58), *p* = 0.001). This significant difference was lost in the long period observation, after the completion of treatment, above all for an increase in heart failure events in the group initially treated with tamoxifen. Women at medium CV profile presented a trend with a consequent significant increase in hospitalizations for heart failure in the aromatase inhibitors group during the 5-year period of treatment (HR = 1.63 (1.21–2.20), *p* < 0.001) and thereafter (HR = 1.42 (1.12–1.81), *p* = 0.004).

## 4. Discussion

The results of our study, conducted on a large cohort of “real life” women over 50 years of age on adjuvant hormone therapy for breast cancer, indicate that, compared to tamoxifen, aromatase inhibitors increase the risk of CV events and, in particular, heart failure. Furthermore, they underscore the importance of coexisting CV risk factors in modulating the effects of hormone therapy for breast cancer on the heart of women in the peri- and post-menopausal period. The protective role of estrogen on the cardiovascular system in terms of favorable lipid profile and vasodilatation is well known [4]. However, as recent experimental evidence has emphasized, they exert a relevant cardioprotective role governing important intracellular processes, such as the intracellular ion flux [18] and the hypertrophic response of cardiomyocytes [19], as well as the collagen synthesis of the extracellular matrix in response to stressors [20]. It is currently assumed that the decline of estrogen at menopause is a major contributor to the pathogenesis of heart failure, particularly the phenotype with preserved ejection fraction, a clinical entity very common in older women with hypertension, central obesity and glycemic derangement [21]. In the current real-world study, we found a trend towards an increased risk of heart failure and CV events in women treated with aromatase inhibitors, a pure anti-estrogen agent, as compared to those treated with tamoxifen, an agonist/antagonist estrogen drug with possibly cardioprotective activity [22,23,24]. During the whole follow-up, aromatase inhibitors were associated with a 20% significant increased risk of heart failure (which was 32% during the 5-year duration of treatment), and 14% increased risk of combined CV events.

These results are consistent with those reported by a meta-analysis of eight RCTs comparing upfront adjuvant aromatase inhibitors to tamoxifen, which showed a 19% (7–34%) increased risk of CV events in women treated with aromatase inhibitors, as well as with those of other “real life” registers reported in the recent literature [25]. A recent study conducted in the UK showed that breast cancer women without previous CV diseases treated with aromatase inhibitors had higher risk of heart failure (HR = 2.80, 1.29 to 6.08) than those treated with tamoxifen. No association was found for myocardial infarction, ischemic stroke, and cardiovascular mortality [8]. No association with myocardial infarction and stroke and anti-aromatase therapy is reported by other observational studies [9,10,11]. Taken together, these results suggest that ischemic heart disease is only one of the drivers leading to the development of heart failure in these patients. Active hormone therapy, indeed, represents a significant risk factor of diabetes among breast cancer survivors [26], particularly aromatase inhibitors, which exert a powerful detrimental effect on insulin sensitivity and central adiposity [27].

In our study, we adopted three different approaches for assessing the association between aromatase inhibitors and CV outcomes. With the intention-to-treat approach, women who started therapy with either aromatase inhibitors or tamoxifen were treated as they were continuously in that exposure group, regardless their exposure during follow-up. However, in our cohort, about 43% of women switched from tamoxifen to aromatase inhibitors, and about 6% from aromatase inhibitors to tamoxifen, so that exposure misclassification may lead to biased results [28]. For this reason, the second approach we used was based on censoring observation at the time of treatment switching (as-treated approach). However, reasons for switching therapies may be different between patients, and patients who switch from one therapy to another may have a different risk of developing the outcome of interest than those who continue the initial therapy. In this case, the resulting informative censoring may lead, again, to biased results [29]. A method to correct for informative censoring is the IPCW, which is the third approach we used in our study. It has been shown that IPCW adjusts for informative censoring by using time-dependent information collected during follow-up, which are likely to affect both censoring (i.e., the switch of therapy) and the occurrence of the study outcomes [16].

Our study has several strengths. First, to our knowledge, this is the largest real-world study assessing the CV risk in almost 34,000 breast cancer women treated with adjuvant aromatase inhibitors or tamoxifen. Second, given the population-based nature of the study, the target population from which the study cohort was selected was representative of the real-world practice. Indeed, all women beneficiaries of the NHS with a diagnostic code of breast cancer during the recruitment period were selected. Third, the IPCW analysis allowed overcoming the bias due to both exposure misclassification and informative censoring.

The present study has also some limitations. First, since patients were not randomly allocated to the treatment with either aromatase inhibitors or tamoxifen, the results may be affected by confounding. Although our analysis considered several measured potential confounders, unmeasured characteristics, such as body mass index, smoking status, menopausal status and physical activity, may affect our conclusions. Furthermore, based on the administrative data, we have no information on the phenotype of heart failure (reduced ejection fraction, preserved ejection fraction or “mid-range” ejection fraction) nor on the incidence/prevalence of asymptomatic dysfunction of the left ventricle. We also do not know if the prescribed therapy adequately controlled hypertension, dyslipidemia, or diabetes. Moreover, our data did not include direct information on biomarkers status, including estrogen receptors, and use of anthracyclines. However, since all women included in our cohort were treated with hormonotherapy, it is likely that all of them were estrogen receptor positive. Moreover, in our cohort, women who received chemotherapy (9% in the PS-matched cohort) did not show higher risk of heart failure (*p* = 0.764) and any CV outcome (*p* = 0.998) as compared to those who did not. Second, despite the high diagnostic accuracy of ICD9-CM codes for detecting CV outcomes, including myocardial infarction and heart failure [30], outcome misclassification cannot be completely excluded in our setting. Finally, despite the large sample size, the study has limited statistical power in order to detect possible differences in the incidence of less common CV outcomes, such as myocardial infarction and ischemic stroke.

## 5. Conclusions

In conclusion, our study offers further evidence that adjuvant therapy with aromatase inhibitors in women with breast cancer aged more than 50 years is associated with increased risk of heart failure and combined CV events, and such therapy probably amplifies the “intrinsic” CV risk of the patient. This underlines the importance of interdisciplinary collaboration between oncologists and cardiologists in evaluating the risk/benefit ratio of the choice of hormone therapy. Considering the efficacy of aromatase-inhibitor hormone therapy against breast cancer, it is necessary to evaluate, by means of prospective studies, whether an appropriate control program for cardiovascular risk factors can improve the prognosis.

## Figures and Tables

**Table 1:** Baseline characteristics (%) of the study cohort.

Characteristic	Original Cohort			Propensity Score Matched Cohort			Weighted Cohort ^¥^		
	AI 26,009	T 7937	SD	AI 7881	T 7881	SD	AI	T	SD
Age, y									
50–60	21.4	44.2	0.50	44.3	44.3	0.00	24.0	32.1	0.18
60–70	36.4	24.4	0.26	24.4	24.4	0.00	37.2	24.2	0.28
≥70	42.2	31.4	0.23	31.3	31.3	0.00	38.8	43.8	0.10
Drug use									
Statins	21.8	14.9	0.18	13.4	15.0	0.05	20.5	19.5	0.03
Anticoagulants	3.7	1.3	0.15	1.4	1.3	0.01	3.2	2.2	0.06
Antidepressants	16.2	15.3	0.02	16.4	15.3	0.03	16.1	16.8	0.02
Antidiabetic	9.9	6.0	0.14	5.5	5.9	0.02	9.2	8.4	0.03
Antihypertensive	57.7	44.5	0.27	42.7	44.5	0.04	55.3	54.3	0.02
Antithrombotic	17.7	12.3	0.15	13.7	12.3	0.04	16.7	16.5	0.01
Bisphosphonates	5.3	6.5	0.05	5.4	6.0	0.03	5.6	6.3	0.03
NSAIDs	40.5	37.0	0.07	36.8	36.9	0.00	40.1	41.0	0.02
Opioids	18.2	15.7	0.07	16.4	15.7	0.02	17.8	18.0	0.01
HRT	5.0	4.7	0.01	4.1	4.8	0.03	5.0	5.2	0.01
Comorbidities									
Peripheral vascular disease	0.1	0.1	0.00	0.1	0.1	0.00	0.1	0.1	0.00
Venous thromboembolism	0.1	0.0	0.04	0.1	0.0	0.04	0.1	0.1	0.00
COPD	0.1	0.1	0.00	0.0	0.1	0.04	0.1	0.1	0.00
Chronic kidney disease	0.3	0.2	0.02	0.4	0.2	0.04	0.3	0.3	0.00
Breast-cancer-related procedure									
Chemotherapy	17.2	9.4	0.23	9.4	9.4	0.00	15.9	13.0	0.08
Radiotherapy	44.1	44.9	0.02	44.3	45.0	0.01	44.3	42.5	0.04

AI: aromatase inhibitors; T: tamoxifen; SD: standardized difference (absolute); COPD: chronic obstructive pulmonary disease; NSAID: nonsteroidal anti-inflammatory drugs; HRT: hormone replacement therapy. ^¥^ Cohort weighted for inverse probability of censoring weights (IPCW) with myocardial infarction as the outcome. Similar characteristics were observed with ischemic stroke, heart failure, and the composite CV outcome.

**Table 2:** Incidence rates of study outcomes in the propensity score matched cohort of women with breast cancer.

Outcome	Aromatase Inhibitors			Tamoxifen			*p* ^§^
	No. of Events	Person-Years	Incidence Rate ^¥^ (95% CI)	No. of Events	Person-Years	Incidence Rate ^¥^ (95% CI)	
Composite CV outcome	473	52,359	9.03 (8.22–9.85)	428	53,767	7.96 (7.21–8.71)	0.029
Myocardial infarction	89	53,344	1.67 (1.32–2.02)	92	54,520	1.69 (1.34–2.03)	0.470
Ischemic stroke	182	53,073	3.43 (2.93–3.93)	163	54,385	3.00 (2.54–3.46)	0.106
Heart failure	266	53,029	5.02 (4.41–5.62)	243	54,355	4.47 (3.91–5.03)	0.097

CI: confidence intervals; ^¥^ per 1000 person-years, based on an intention-to-treat (ITT) approach; ^§^ *p*-value of the test homogeneity of incidence rates between groups.

**Table 3:** Association between hormonotherapy (aromatase inhibitors vs tamoxifen) and study outcomes.

Analysis	ITT	As-Treated	IPCW ^¥^
Outcome	HR (95% CI)	HR (95% CI)	HR ^§^ (95% CI)
Composite CV outcome	1.14 (1.00–1.30)	1.07 (0.92–1.24)	1.14 (1.00–1.29)
Myocardial infarction	0.99 (0.74–1.33)	1.03 (0.74–1.44)	0.97 (0.74–1.28)
Ischemic stroke	1.15 (0.93–1.42)	1.06 (0.83–1.35)	1.07 (0.87–1.31)
Heart failure	1.13 (0.95–1.35)	1.03 (0.85–1.25)	1.20 (1.02–1.42)

ITT: intention-to-treat; IPCW: inverse probability of censoring weights; CI: confidence intervals; ^¥^ cohort weighted for IPCW; ^§^ adjusted by age.

## Data Availability

The data that support the findings of this study are available from Lombardy Region, but restrictions apply to the availability of these data, which were used under license for the current study, and so are not publicly available. Data are however available from the Lombardy Region upon reasonable request.

## Supplementary Materials

The following are available online at https://www.mdpi.com/article/10.3390/cancers13092254/s1, Table S1: ICD-9 CM and ATC codes of diseases/conditions and medicaments/drugs used for the current study.
