# Supplementary material for: Adjuvant Hormonotherapy and Cardiovascular Risk in Post-Menopausal Women with Breast Cancer: A Large Population-Based Cohort Study
Source: Cancers (Basel). 2021 May 8;13(9):2254. doi: 10.3390/cancers13092254 (PMC8125834; doi:10.3390/cancers13092254)
Supplement: Supplementary file 1 [file cancers-13-02254-s001.zip › cancers-1193531-supplementary.pdf]

Table S1. ICD-9 CM and ATC codes of diseases/conditions and medicaments drugs used for the current study.

Disease/condition	ICD9-CM codes
Breast cancer	174
Any malignant cancer	140–208
Breast cancer surgery	85.20–85.25, 85.33–85.36, 85.41–85.48
Distant metastases	197, 198, 199.0
Myocardial infarction	410
Congestive heart failure	428
Ischemic stroke	433–434, 436
Peripheral vascular disease	443
Venous thromboembolism	453
Chronic obstructive pulmonary disease	490–492, 494, 496
Chronic kidney disease	585, 586, 584.5–584.9
Chemotherapy	V58.1, 99.25, 99.28
Radiotherapy	922, V58.0
**Drug**	**ATC codes**
Antineoplastic drugs	L01
Aromatase inhibitors	L02BG03, L02BG04, L02BG06
Tamoxifen	L02BA01
Anticoagulants	B01AA, B01AE, B01AF
Antidepressant	N06
Antidiabetic	A10
Antihypertensive	C02, C03, C07, C08, C09
Statins	C10
Antithrombotic	B01AC
Bisphosphonates	M05BA, M05BB
Non-steroidal anti-inflammatory drugs (NSAIDs)	M01A, M01B
Opioids	N02A
Hormone replacement therapy	G03C
